# The Impact of Body Mass Index on Short- and Long-Term Outcomes in Patients Undergoing Coronary Artery Graft Bypass

**DOI:** 10.1371/journal.pone.0095223

**Published:** 2014-04-21

**Authors:** Hushan Ao, Xianqiang Wang, Fei Xu, Zhe Zheng, Ming Chen, Lei Li, Chaoqun Wu, Qian Wang, Shengshou Hu

**Affiliations:** Department of Anesthesiology, State Key Laboratory of Translational Cardiovascular Medicine, Fuwai Hospital & Cardiovascular Institute, Chinese Academy of Medical Science, Peking Union Medical College, Beijing, China; Thomas Jefferson University, United States of America

## Abstract

**Objective:**

This study was designed to investigate the impact of body mass index (BMI) on short- and long-term outcomes after initial revascularization with coronary artery bypass graft (CABG) surgery.

**Methods:**

4916 Chinese who consecutively underwent isolated, primary CABG at the Cardiovascular Institute of Fuwai Hospital from January 1, 1999 to December 31, 2005 were included in this study. They were classified based on BMI as follows: underweight: <18.5 kg/m^2^, normal weight: 18.5 to 23.9 kg/m^2^, overweight: 24 to 27.9 kg/m^2^, obesity: 28 to 32 kg/m^2^, and severe obesity: >32 kg/m^2^. Short (in-hospital) and long-term (5-years) major post-operative complications and mortalities were compared among various BMI groups after initial revascularization.

**Results:**

Multiple regression analysis of five years follow-up of clinical end points indicated that various BMI groups were not associated with significant differences in 5 years mortality and MACCE, however, old age, smoking, hypertension, myocardial infarction and heart failure were the risk factor for the mortality.

**Conclusions:**

In this large-scale study with long term follow-up after primary CABG in an exclusively ethnic Chinese population, we found that different BMI groups were not significantly associated with 5-years mortality and MACCE, however, old age, smoking, hypertension, myocardial infarction and heart failure were the risk factors of post-operative mortality, and old age, hypertension and heart failure increased the rate of MACCE.

## Introduction

Obesity is a common and growing health problem; almost one third of American adults are obese [1]. Annual health care costs attributable to obesity have been estimated to be approximately $68 billion, with an additional $30 billion being spent on weight-reduction programs and special diet [1]. The relationship of obesity to long-term survival is complex and most studies have found a J- or U-shaped curve with increasing mortalities in the underweight or very obese [2], [3]. However, when the data have been adjusted for smoking and concurrent illness, the relationship has been more linear and the risk of death rises as body mass index (BMI) increases [4]. BMI is a validated measure of adiposity [5] and has been consistently used to analyze of obesity and mortality. Obese adults are at an increased risk of cardiovascular mortality [6]. These studies have been limited by failing to account for important confounding factors, such as smoking and comorbidity. Furthermore, no long-term data are available on the impact of BMI on survival in a large series of patients underwent coronary artery bypass graft (CABG) surgeries.

Studies have identified factors that contribute to balance in preventing preoperative adverse events, and potentially improving quality of life [7]. Body fat (commonly described by BMI) in CABG patients is an independent risk factor for blood loss: very low BMI (<18.5 kg/m^2^) has been identified as increasing blood loss [8]–[10].

This study retrospectively evaluated the effects of BMI on short and long term outcomes in Chinese patients who underwent isolated, primary CABG [11]–[13].

## Methods

### Study Population

This was a retrospective, observational study of consecutive patients who underwent isolated primary CABG at the Fuwai Hospital. The study protocol was approved by the the Ethics Committee of Fuwai Hospital, and the written informed consent was waived. 4916 patients who consecutively underwent isolated, primary CABG from January 1, 1999 to December 30, 2005 at Fuwai Hospital in Beijing, China were included in this study. BMI was defined as weight, in kilogram, divided by height in meter squared. As the current World Health Organization's (WHO) definition of adult overweight (BMI>25 kg/m^2^) and obesity (BMI>30 kg/m^2^) may not be applicable to the Chinese population, thus it had been suggested that the definition of using BMI for overweight or obesity for the Chinese population is different from that for the North American or European populations because obesity-associated metabolism is lower in Chinese individuals than in North American or European populations [14]. Therefore, we adopted the Chinese BMI definition proposed by the Working Group on Obesity in China (WGOC) [15] and described in the Guidelines for Prevention and Control of Overweight and Obesity in Chinese Adults [16]to define overweight or obesity, as follows: BMI<18.5 kg/m^2^ (underweight), 18.5≤BMI<24 kg/m^2^ (normal weight), 24≤BMI<28 kg/m^2^ (overweight), and BMI≥28 kg/m^2^ (obese). To investigate the severe obese in this study, we modified that defined as underweight (<18.5 kg/m^2^), normal weight (BMI 18.5 to 23.9 kg/m^2^), overweight (BMI 24 to 27.9 kg/m^2^), obesity (BMI 28 to 32 kg/m^2^), and severe obesity (BMI>32 kg/m^2^).

Isolated primary CABG was defined as coronary artery bypass graft surgery alone for the first time with or without cardiopulmonary bypass. Patients who underwent combined cardiac surgical procedures were excluded. Data were collected from hospital medical records.

### Outcome events definition

Renal failure was defined as a need for dialysis to treat prolonged oliguria or anuria [17]; stroke as central neurological deficit persisting more than 72 h; coma as being unresponsive for more than 24 h; encephalopathy as reversible neurological deficit (recovery within 72 h of onset); low cardiac output syndrome (LCOS) as cardiac index lower than 2.0 l/min per m^2^ and left ventricular assist (LVAD), intra-aortic balloon pump (IABP) and inotropic support after surgery. Outcomes were recorded from follow-up included death and major adverse cardiac and cerebrovascular events (MACCE). MACCE was defined as permanent or transient stroke, coma, perioperative myocardial infarction (MI), heart block, and cardiac arrest [18]–[20].

### Statistical analysis

SPSS version 17.0 (SPSS Inc., Chicago, IL, USA) was used for statistical analysis. Patient demographics, perioperative and follow up outcomes were analyzed. Demographic data are summarized in Tables 1 and 2. Continuous parameters are described by mean and standard deviations, and frequencies describe categorical variable. Comparisons of numerical variables between two groups were performed with Student's test or Mann–Whitney U test if not meeting the normal distribution. ANOVA was used to compare numerical variables between more than two groups. Comparisons of categorical variables were performed with Chi-square tests. Correlations between variables were measured using Spearman rank correlation coefficient. Cumulative incidence of mortalities and MACCE of each BMI groups at 5 year were estimated by Kaplan-Meier method and compared by log-rank test. Multiple Cox regression model and multiple logistic regression model were used to analysis follow-up mortalities and MACCE separately. Covariates included in multiple regression models were baseline variables that showed significant difference among 5 BMI groups. A two-tailed P value <0.05 was considered as statistically significance.

**Table 1:** Baseline Characteristics of the Patients in All Five BMI Groups.

Variable	BMI(kg/m^2^)						
	<18.5	18.5–23.9	24–27.9	28–32	>32	Total	P-value
	(n = 31)	(n = 1387)	(n = 2496)	(n = 849)	(n = 153)	(n = 4916)	
Age(yrs)	67.1±6.2	61.8±8.4	59.6±8.5	58.8±8.7	59.2±8.6	60.1±8.6	<.0001
BMI(kg/m^2^)	17.4±1.0	22.3±1.3	25.9±1.1	29.4±1.1	50.5±49.8	26.2±10.1	<.0001
Men	20(64.5%)	1110(80.0%)	2133(85.5%)	729(85.9%)	123(80.4%)	4115(83.7%)	<.0001
Smoking	10(32.3%)	672(48.5%)	1348(54.0%)	496(58.4%)	86(56.2%)	2612(53.1%)	<.0001
Family History	1(3.2%)	107(7.7%)	206(8.3%)	63(7.4%)	6(3.9%)	383(7.8%)	0.2869
Hypertension	15(48.4%)	784(56.5%)	1535(61.5%)	571(67.3%)	107(70.0%)	3012(61.2%)	<.0001
Hyperlipidemia	8(25.8%)	480(34.6%)	936(37.5%)	345(40.6%)	69(45.1%)	1838(37.4%)	0.0070
Diabetes Mellitus	7(22.6%)	388(28.0%)	633(25.4%)	205(24.2%)	36(23.5%)	1269(25.8%)	0.2427
History of Renal Failure	0	17(1.2%)	16(0.6%)	3(0.4%)	0	36(0.7%)	0.0929
Creatinine (µmol/L)	193.0±328.9	103.5±101.5	109.1±111.0	122.2±161.9	104.8±154.8	110.0±130.0	0.1180
Cerebrovascular Events	5(16.1%)	94(6.8%)	163(6.5%)	45(5. 3%)	8(5. 2%)	315(6. 4%)	0.1183
Peripheral Artery Disease	6(19. 4%)	152(11.0%)	212(8. 5%)	58(6.8%)	18(11.8%)	446(9. 1%)	0.0016
Thrombolytic Therapy	3(9.7%)	133(9.6%)	248(10.0%)	56(6.6%)	8(5.2%)	488(9.1%)	0.0196
Unstable Angina Pectoris	1(3.2%)	152(11.0%)	234(9.4%)	87(10.3%)	24(15.7%)	498(10.1%)	0.1186
Myocardial Infarction	11(35.5%)	682(49.2%)	1229(49.2%)	397(46.8%)	73(47.7%)	2392(48.7%)	0.4177
Diseased Coronary Artery	2.9±0.2	2.8±0.5	2.8±0.5	2.8±0.5	2.8±0.5	2.8±0.5	0.4558
Left Main Disease	8(25.8%)	504(36.3%)	700(28.0%)	230(27.1%)	35(22.9%)	1477(30.0%)	<.0001
Heart Failure	1(3.2%)	37(2.7%)	45(1.8%)	9(1.1%)	6(4.0%)	98(2.0%)	0.0311
Atrial Fibrillation	0	41(3.0%)	38(1.5%)	17(2.0%)	5(3.3%)	101(2.1%)	0.0278
LVEF (%)	59.4±9.7	60.0±10.1	59.7±9.4	59.2±9.1	58.2±8.9	59.5±9.5	0.2901
Intravenous Nitrate	0.06±0.2	0.06±0.2	0.05±0.2	0.06±0.2	0.09±0.3	0.06±0.2	0.1968

BMI: body mass index; LVEF: left ventricular ejection fraction. LVEF: left ventricular ejection fraction; ANOVA test was used to analyze continuous variables, χ2 test for categorical variables. P<0.05 was accepted as statistically significant.

**Table 2:** Post-operative Characteristics of All Five BMI Groups.

Variable	BMI(kg/m^2^)						
	<18.5	18.5–23.9	24–27.9	28–32	>32	Total	P-value
	(n = 31)	(n = 1387)	(n = 2496)	(n = 849)	(n = 153)	(n = 4916)	
Postoperative Complications	0	4(0.3%)	14(0.6%)	2(0.2%)	0	20(0.4%)	0.5076
Epinephrine	7(22.6%)	181(13.1%)	246(9.9%)	109(12.8%)	14(9.2%)	557(11.3%)	0.0030*
Stroke	0	4(0.3%)	9(0.4%)	4(0.5%)	2(1.3%)	19(0.4%)	0.4010
Duration of ICU Stay (h)	69.4±74.7	63.4±61.3	59.9±57.8	60.3±53.6	65.5±60.2	61.2±58.3	0.3025
Ventilation Time (h)	14.8±7.6	17.6±32.2	16.1±21.4	16.4±17.6	18.6±28.4	16.7±24.6	0.3841
Atrial Fibrillation	3(9.7%)	133(9.6%)	215(8.6%)	56(6.6%)	14(9.2%)	421(8.6%)	0.1858
Renal Failure	0	6(0.4%)	6 (0.2%)	2(0.2%)	0	14(0.3%)	0.7655
Coma	0	7(0.5%)	9(0.4%)	3(0.4%)	0	19(0.4%)	0.8639
Mortality	2(6.5%)	62(%4.5)	102(4.1%)	37(4.4%)	7(4.6%)	210(4.3%)	0.9446
Myocardial Infarction	0	19(1.4%)	37(1.5%)	14(1.7%)	3(2.0%)	73(1.5%)	0.9119
Repeated Revascularization	3(9.7%)	48(3.5%)	110(4.4%)	44(5.2%)	4(2.6%)	209(4.3%)	0.1191
Stroke	6(19.4%)	172(12.4%)	285(11.4%)	102(12.0%)	22(14.4%)	587(11.9%)	0.4906
MACCE	11(35.5%)	287(20.7%)	498(20.0%)	183(21.6%)	32(20.9%)	1011(20.6%)	0.2549

MACCE: major adverse cardiac and cerebrovascular events. BMI: body mass index; ICU: intensive care unit; Student's t test was used to analyze continuous variables, χ2 test for categorical variables. P values: comparison between overweight and control groups; * statistical differences between BMI<18.5 group and the rest of the BMI groups. P < 0.05 was considered statistically significant.

## Results

### Baseline characteristics

Table 1 shows patient demographic data. Underweight group tended to be older (67.1±6.2) than patients in other groups. A higher number of underweight patients presented as emergency cases, whereas more elective procedures were performed in the obese group. Hyperlipidemia was more prevalent in all the obese groups (45.1% vs. 25.8%, 34.6%, 37.5%, 40.6%, 37.4% respectively, p<0.01), while peripheral and cerebrovascular disease prevailed in the lean cohort (peripheral artery disease: 19. 4% vs. 11.0%, 8. 5%, 6.8%, 11.8%, 9.1%; cerebrovascular events:16.1% vs. 6.8%, 6.5%, 5. 3%, 5. 2%, 6.4%. p<0.05). Renal function was similar across the population.

### Perioperative outcomes

Table 2 shows the results of the five groups. Among all 5 groups, there are no differences in the in-hospital postoperative complications (P = 0.051). Patients in underweight group administered more epinephrine compared with other groups (22.6% vs. 13.1%, 9.9%, 12.8%, 9.2%, 11.3% respectively. P<0.05). There was no difference in in-hospital mortality. There were no differences in stroke, length of ICU stay, ventilation time, atrial fibrillation, renal failure, composite cerebral complication, and coma (p>0.05).

**Figure 1:**
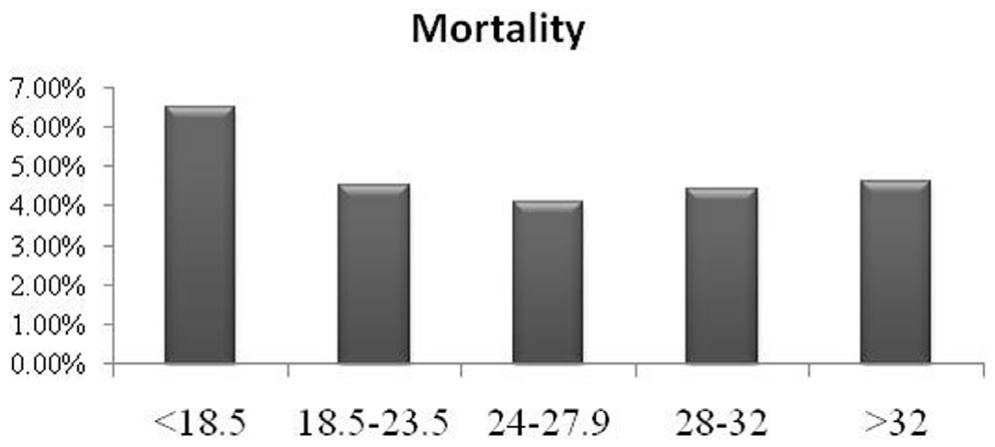
Illustrating the mortality rates at the end of 5-year follow-up in all five body mass index (BMI) groups.

**Figure 2:**
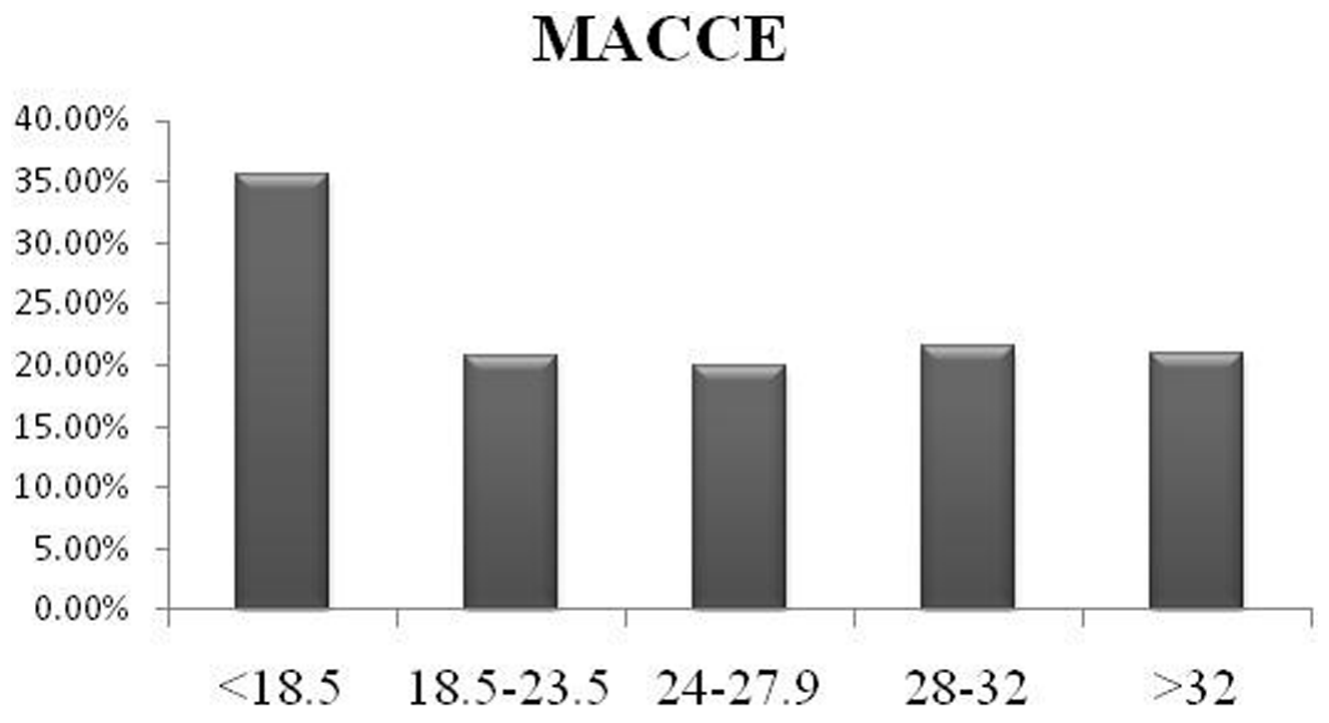
Illustrating the major adverse cardiac and cerebrovascular events (MACCE) rates at the end of 5-year follow-up in all five body mass index (BMI) groups.

**Figure 3:**
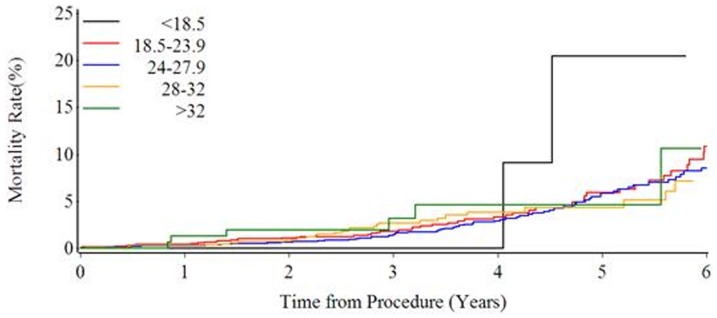
Kaplan-Meier curves of the 5-year mortality in all five body mass index (BMI) groups.

The median follow up duration was 59.3 months in this population. There were no significant differences in myocardial infarction, stroke and revascularization rate (p>0.05). There were also no significant differences in mortality (p>0.05) and MACCE (p>0.05). However, the mortality and MACCE tend to be higher in underweight group than that in other groups, though no significant difference was found (mortality: 6.5% VS. 4.5%, 4.1%, 4.4%, 4.6%, 4.3% respectively; MACCE: 35.5% vs. 20.7%, 20.0%, 21.6%, 20.9%, 20.6% respectively P>0.05; Figure 1–2). Figure 3 shows the Kaplan-Meier curves demonstrated the mortality, and there was no significant difference among 5 groups (Log-rank test P = 0.7909).

The long-term follow-up mortality: Multivariable Cox regression model was used to adjust the confounders and analysis the association between BMI groups and mortality. Covariates in this model included age, BMI, gender, smoking, hypertension, hyperlipidemia, peripheral artery disease, MI, thrombolytic therapy, left main coronary artery disease, heart failure. As demonstrated in Table 3, old age, smoking, hypertension, MI and heart failure are associated with increased long-term follow-up mortalities [Hazard Ratio (HR)(95% CI):1.053(1.035–1.072), 1.464(1.082–1.980), 1.751(1.292–2.372),1.394(1.034–1.880), 2.817(1.544–5.140).p<0.05]. There were no significant differences found in all 5 BMI groups (p>0.05).

**Table 3:** Long Term Mortality.

Variables	β	std	P-value	HR	HR 95% CI	
BMI <18.5 vs. 18.5–23.9	0.23779	0.72457	0.7428	1.268	0.307	5.248
BMI 24–27.9 vs. 18.5–23.9	−0.03860	0.16625	0.8164	0.962	0.695	1.333
BMI 28–32 vs. 18.5–23.9	−0.07927	0.21944	0.7179	0.924	0.601	1.420
BMI>32 vs. 18.5–23.9	0.34369	0.40349	0.3943	1.410	0.639	3.110
Age	0.05208	0.00904	<.0001	1.053	1.035	1.072
Gender: Male vs. Female	0.35880	0.24604	0.1448	1.432	0.884	2.319
Smoking	0.38097	0.15420	0.0135	1.464	1.082	1.980
Hypertension	0.55994	0.15509	0.0003	1.751	1.292	2.372
Hyperlipidemia	−0.13044	0.15397	0.3969	0.878	0.649	1.187
Peripheral Artery Disease	0.39404	0.28771	0.1708	1.483	0.844	2.606
Thrombolytic Therapy	−0.01460	0.22105	0.9473	0.986	0.639	1.520
Myocardial Infarction	0.33206	0.15257	0.0295	1.394	1.034	1.880
Heart Failure	1.03561	0.30684	0.0007	2.817	1.544	5.140
Left Main Disease	0.10601	0.14930	0.4777	1.112	0.830	1.490

BMI: body mass index; Multivariable Cox regression model was used to analyze categorical variables. β: std: standard deviation; HR: hazard ratio; CI: confidence interval. P values: comparison between the other and normal weight group; P<0.05 was considered as statistically significant.

The 5 years follow-up MACCE: Multivariable logistic regression model was used to adjust the confounding factors and analysis the association between BMI groups and MACCE. Table 4 showed old age, hypertension and heart failure are associated with increased the rate of MACCE [HR (95% CI): 1.026(1.017–1.035), 1.254(1.080–1.455), 1.583(1.014–2.472). p<0.05], while there were no significant associations between BMI groups and MACCE (p>0.05).

**Table 4:** Major Adverse Cardiac and Cerebrovascular Events (MACCE) at the End of Five Years.

Variables	β	std	P-value	OR	OR 95% CI	
BMI<18.5 vs. 18.5–23.9	0.5252	0.3076	0.0878	1.997	0.939	4.248
BMI 24–27.9 vs. 18.5–23.9	−0.1738	0.0974	0.0744	0.993	0.841	1.172
BMI 28–32 vs. 18.5–23.9	−0.0677	0.1111	0.5420	1.104	0.891	1.367
BMI>32 vs. 18.5–23.9	−0.1173	0.1790	0.5124	1.050	0.694	1.591
Age	0.0257	0.00445	<.0001	1.026	1.017	1.035
Gender: Male vs. Female	0.0165	0.0525	0.7537	1.034	0.841	1.270
Smoking	0.0700	0.0388	0.0715	1.150	0.988	1.339
Hypertension	0.1130	0.0380	0.0030	1.254	1.080	1.455
Hyperlipidemia	−0.0150	0.0371	0.6855	0.970	0.839	1.122
Peripheral Artery Disease	−0.0965	0.0638	0.1302	0.824	0.642	1.059
Thrombolytic Therapy	0.0389	0.0634	0.5395	1.081	0.843	1.386
Myocardial Infarction	0.0391	0.0375	0.2971	1.081	0.934	1.252
Heart Failure	0.2297	0.1137	0.0434	1.583	1.014	2.472
Left Main Disease	0.00983	0.0389	0.8003	1.020	0.876	1.188

BMI: body mass index; multivariable logistic regression model was used to analyze categorical variables; β: std: standard deviation; HR: hazard ratio; CI: confidence interval. P values: comparison between the other and normal weight group; P<0.05 was accepted as statistically significant.

## Discussion

Obesity is a common and increasingly prevalent health issue throughout the world. It has been associated with the development of diabetes mellitus, hypertension, cardiovascular disease and heart failure [21], [22]. The relation between BMI and surgical outcomes is complex [23].Recent studies have described “obesity paradox” phenomena [11], [24]–[29]. Despite the association of obesity with chronic disease that lead to early death, improved survival has been observed in obese patients with heart failure and following CABG surgery [30], [31]. Obesity was found to be a significant factor associated with smaller infarction size following MI. However, most of these reports were in the western populations.

This is the first and the largest study to date in examining outcomes after CABG surgery in the Chinese population, stratified according to BMI. In this study, the worse outcomes were seen in patients at the body mass with a BMI<18.5 kg/m^2^, although this accounted for only 0.63% of the sample size that is consistent with previous reports in western population [2], [3]. Compared with obese patients, underweight patients in this series had a higher rate of epinephrine administration and MACCE that somehow suggesting that underweight weight is a possible risk factor in patients with isolated CABG.A previous report indicated that fat mass is the main energy storage of the body [32], supply energy in underweight patients undergoing CABG may confer survival advantages.

We found no significant differences in in-hospital mortality in all 5 groups. However, although 5-years mortality rate tend to be higher in underweight group than other groups, there was no statistically significant difference seen among all 5 groups. Thus the present study suggested a possible link between the underweight and the mortality, which has been showed in previous studies [33], [34]. Further studies, especially large sample size of the underweight patients are needed to confirm these findings. Because there was no BMI>40 kg/m^2^ available in our database that may due to racial differences, we are unable to find out the mortality in extreme severe obese patients in Chinese patients.

Traditionally, obesity has been viewed as a risk factor for postoperative mortality in patients undergoing CABG surgery [10]. Several studies have explored this potential association reported with mixed results. Some studies found a higher incidence of mortality in the postoperative and mid-term follow-up after CABG in obese patients [24]–[27], whereas others did not [12], [13], [30], [31], [35]–[41]. There are reports of increased operative mortality in morbidly obese individuals [11], [28]. We did not find a significant association between obesity and mortality either immediately after surgery or at the end of 5-year follow-up even after multivariable Cox regression analysis. However, we did find that old age, smoking, hypertension, MI and heart failure were the major risk factors for long-term mortality, and old age, hypertension and heart failure increased the rate of MACCE in ethnic Chinese population after CABG surgery. The inconsistent results between our research and prior ones might be caused of the following aspects: Firstly, our objects is the Chinese, unlike previous white people, where may exist racial differences. The classification criteria of BMI we used also reflected this difference (we use China's national standard which is different from the WHO criteria), and this is also the second reason why our results cannot be directly used to compare prior ones; secondly, unlike some multi-center study, this study is a single center study. However, these factors associated with 5 years mortalities were consistence with Nalysnyk et al. Meta-analysis reports where found that old age, hypertension, and history of prior heart surgery and MI are associated with increased risk of death after CABG [42].

Several statistical methods were applied in this study to test and adjust for bias between the five groups. Multivariable logistic and Cox regression analyses were used to examine whether normal weight was independently related to defined postoperative outcomes. As a result, the derived five groups showed no difference in baseline characteristics and had identical results as the unmatched analysis.

Studies in cardiovascular surgery focused largely on CABG have found mixed results. Jin, et al, postulated that this was due to different BMI classifications and sample sizes [12]. However, what is intriguing about Jin's study was their finding of reduced mortality in mildly obese patients. Similarly, Romero-Corral, in a meta-analytical review, found that overweight CAD patients had the lowest risk of cardiovascular complications and overall mortality. These data differed from what we found in Chinese patients, which may be due to the difference in race(need provide reference number).

Our finding was different from others that higher incidence of renal insufficiency and diabetes mellitus occurs in obese patients [43], [44]. We found no difference in ICU stay in the postoperative period in obese patients compared with non-obese patients that consistent with other repots [45], [46].

In addition, while it is widely accepted that obesity increases the risk of heart disease, a growing number of recent reports document a significant survival benefit in obese patients once they have been diagnosed. This has been termed the “obesity paradox”. The current study supports this observation by demonstrating lower mortality and lower vasoactive medicine requirement in normal weight and mild to moderate obese patients and raises the question why mild to moderate obese patients have better short term outcomes than long term outcomes.

### Study limitations

The current study did not show a significant difference in the mortality among different BMI groups, although a trend to increase in the mortality and a higher vasoactive medicine requirement were seen in the underweight group. It needs further studies on the relationship between BMI and outcomes in patients undergoing CABG in the Asian population.

## Conclusion

This large-scale study with long term follow-up after primary CABG in an exclusively ethnic Chinese population found that there were no significant associated with 5-year mortality and MACCE with different BMI groups, however, old age, smoking, hypertension, MI and heart failure were the risk factors of follow-up mortality, and old age, hypertension and heart failure increased the rate of MACCE.
